# Community based study to assess the prevalence of diabetic foot syndrome and associated risk factors among people with diabetes mellitus

**DOI:** 10.1186/s12902-018-0270-2

**Published:** 2018-06-26

**Authors:** S. P. Vibha, Muralidhar M. Kulkarni, A. B. Kirthinath Ballala, Asha Kamath, G. Arun Maiya

**Affiliations:** 1 Department of Community Medicine, Kasturba Medical College, Manipal Academy of Higher Education, Manipal, Karnataka 576104 India; 2 Department of Statistics, Prasanna School of Public Health, Manipal Academy of Higher Education, Manipal, Karnataka 576104 India; 3 Department of Physiotherapy, School of Allied Health Sciences, Manipal Academy of Higher Education, Manipal, Karnataka 576104 India

**Keywords:** Diabetic foot syndrome, Diabetic peripheral neuropathy, MNSI, Prevalence, Community based study

## Abstract

**Background:**

Diabetic foot is one of the most significant and devastating complication of diabetes. The objective of this study was to assess the prevalence of diabetic foot syndrome (DFS) and the associated risk factors among people with diabetes mellitus.

**Methods:**

A community based cross-sectional study was carried out among 620 subjects with diabetes mellitus (DM) in rural areas of Udupi district. The Michigan Neuropathy Screening Instrument was used to identify peripheral neuropathy. Ankle brachial index was used to identify peripheral arterial disease (PAD). Subjects with diabetic foot syndrome were classified according to the International Working Group on Diabetic Foot (IWGDF) classification system.

**Results:**

The overall prevalence of DFS was 51.8%. Among them 31.3, 11.9 and 8.5% belonged to category 1, 2 and 3 respectively. Multivariate logistic regression analysis showed advancing age, low socio-economic status, sedentary physical activity and longer duration of DM were significant independent correlates of DFS.

**Conclusion:**

The overall prevalence of DFS was high among the study population; hence the screening for foot complications should start at the time of diagnosis of diabetes integrated with sustainable patient education at primary care level by training of health care providers at primary care level.

## Background

Diabetes mellitus is a major public health problem with rising prevalence worldwide and in the year 2015 around 415 million people were known to have diabetes. This estimate is expected to increase to 642 million of the population by 2040 [1]. Further, it is the 6th leading cause of death [2], attributing to 5 million deaths globally in 2015. According to recent estimates, 69.2 million people are affected with diabetes in India [1].

Along with the raising prevalence of diabetes, an increase in its complications is also expected. Diabetes along with its complications is expected to result in increasing morbidity, mortality and health expenditure due to the requirement of specialized care [3].

Diabetic foot is one of the most significant and devastating complication of diabetes and is defined as a group of syndromes in which neuropathy, ischemia and infection lead to tissue breakdown, and possible amputation [4]. Around 15% of diabetic patients will develop foot ulcers in their life time and this is known to precede amputation in 85% of the cases [5]. Every 20 s a lower limb is lost to diabetes in the world and it is the most common cause of non-traumatic lower limb amputation [6]. It is estimated that approximately 45,000 lower limbs are amputated every year in India and the vast majority of these are probably preventable [5].

Prevention of diabetic foot ulceration is critical in order to reduce the associated high morbidity and mortality rates, and the danger of amputation. A number of contributory factors work together to cause foot ulceration in patients with diabetes. These include peripheral neuropathy; mechanical stress and peripheral vascular disease [7].

Regular comprehensive foot examination, patient education on foot care like simple hygienic practices, provision of appropriate footwear, and prompt treatment of minor injuries and a multi-disciplinary team approach can decrease ulcer occurrence by 50% and amputations by up to 85%. [3, 8].

Identification of diabetics with DFS and its associated factors is the key to reduce further complications and to have baseline information to initiate appropriate interventions. There is a dearth of community based studies in Coastal Karnataka, which assess the prevalence of diabetic foot syndrome and associated risk factors among diabetics. Hence the present study was planned to find the prevalence of foot problems and determine the risk factors leading to DFS.

## Methods

Approval of institutional ethics committee was taken prior to conducting the study. All reported cases of diabetes mellitus aged more than 18 years, residing at least for the past 1 year in the study area were included in the study. Patients with gestational diabetes mellitus, stroke, bilateral below knee amputation, Hansen’s disease and foot deformities secondary to other causes were excluded.

This is a community based cross-sectional study carried out during August 2015 to September 2017 among reported cases of diabetes mellitus currently residing in field practice area of Department of Community Medicine, Kasturba Medical College (KMC), Manipal. It is situated along the coastal belt of Udupi District of Karnataka state, India covering a population of 45,246 spread out over 13 villages. The healthcare services are provided by both public and private sectors. The area has good collaboration between two sectors with primary, secondary and tertiary care facilities in the vicinity. These villages have a homogenous population in terms of occupation, SES and food habits. The detailed information of the population in the field practice area is captured and fed into the central database in e-RMCWH portal which can be accessed any time.

According to previous study done by George H [9] et al. the prevalence of peripheral neuropathy, a component of DFS, among people with diabetes was reported to be 47%. Thus, with 10% precision on prevalence of 47%, sample size of 433 subjects was obtained. Considering 40% non-response, sample size was estimated to be 721. The list of all diabetes patients residing at field practice area was obtained from e- database. Complete enumeration of reported cases of diabetes in a given locality was done, those available at the time of survey were included and adjacent locality was selected till the sample size was met.

The identification of households having patients with diabetes in the community was done with the help of health worker. A home visit was conducted and the purpose of the visit was explained. An informed written consent was taken. Subjects were interviewed using pre-structured questionnaire to collect data on socio-demographic details, history of diabetes mellitus including treatment and associated risk factors for development of diabetic foot including dietary habits, physical activity [10], tobacco use and alcohol consumption [11].

Physical activity was assessed according to a survey questionnaire used by Ramachandran et al. [10]**.** This tool is validated for Indian settings and uses a scoring system to grade the physical activity. Four categories of occupation are considered. (i) Manual labourers (including masons, carpenters and those who carry loads, agricultural work, e.g. ploughing and tilling); (ii) Office jobs or desk work; (iii) Housewives and retired persons; (iv) Persons unable to work. Duration of activities for each day and number of working days in a week were considered to calculate the score which gives a minimum score of one and maximum of 70. Based on the scores physical activity was graded as sedentary (score: 1–17); light (score: 18–34); moderate (score: 35–51) and strenuous (score: > 51).

Anthropometric measurements were noted as per WHO standard guidelines [12]. Blood pressure of the subject was measured as high blood pressure is considered as a risk factor for diabetic foot syndrome [13].

Michigan Neuropathy Screening Instrument (MNSI) [14] was used to screen for diabetic peripheral neuropathy. It had two components, the history and the physical assessment. The first part of the screening instrument comprises of 15 self-administered “yes or no” questions on foot sensation including pain, numbness, and temperature sensitivity. A higher score (out of a maximum of 13 points) indicates more neuropathic symptoms. The second part of the MNSI is a brief physical examination involving 1) inspection of the feet for deformities, dry skin, hair or nail abnormalities, callous, or infection; 2) semi-quantitative assessment of vibration sensation at the dorsum of the great toe; 3) grading of ankle reflexes; and 4) monofilament testing. Patients screening positive on the clinical portion of the MNSI (greater than 2.5 points on a 10 point scale) were considered neuropathic.

Vascular assessment [15] of feet was done by manual assessment of foot pulses in both lower limbs for posterior tibial and dorsalis pedis pulses and manual measurement of ankle-brachial index (ABI). Absence of peripheral pulses and ABI ≤ 0.9 was considered as peripheral arterial disease (PAD) [16].

The subjects found to be having foot problems were classified according to The International Working Group on Diabetic Foot (IWGDF) Risk Classification System [15]. Glycated hemoglobin (HbA1c) was estimated in sub sample population, taking equal number of subjects with foot at risk category 0, 1, 2 and 3 as identified from the survey matched for age and gender**.** Health education regarding foot care practices was given to all subjects. If the subjects were found to be category 1 or 2 were referred to nearest RMCW home for timely screening and subjects with category 3 risk were referred to Diabetic Foot clinic at KMC, Manipal.

The collected data was tabulated and analysed by using software SPSS (Statistical Package for Social Sciences) V.15.0 (SPSS South Asia, Bangalore) for windows. The data was cross checked for data entry errors. Findings were described in terms of proportions and their 95% confidence intervals. Continuous data was summarized using mean, and standard deviation or median and inter quartile range depending on skewness of data. Chi-square test was used to find the association and *p*-value < 0.05 was considered significant. Multiple logistic regression was used to find the risk factors.

## Results

### Socio-demographic details

In the study, there was a favourable response and the non-response rate was 13.7% with 620 diabetics consenting to participate in the study. The mean age of the participants was 63.37 years (SD 10.8) with 61.2% of subjects being in the age group above 60 years. There was female preponderance (57.4%) and majority of the study participants were Hindus. An overall literacy rate among study participants was 85.8%, with 57.6% having education up to high school. Housewives accounted for 46.9% of the study participants, skilled workers 11.7 and 10.8% were currently unemployed and 9.5% were retired.

SES was assessed as per modified Udai Pareek scale and majority (70%) of the study population belonged to middle class, 28.1% to low class and only 1.6% belonged to high class.

### Details of DM and health seeking behaviour

Of the 620 participants, all were having type 2 DM with median duration of 7 years (IQR 3, 13) and 42.4% of the study population was diagnosed within the last 5 years. Half (53.2%) of the participants gave a family history of DM and only 6.3% gave a history of foot complications related to DM among their first degree relatives.

Majority (96.4%) were on allopathic treatment, among them 89.6% were on oral hypoglycemic agents (OHAs), 3.5% were on insulin and rest 6.8% were on combination of OHAs and insulin. Over 96.1% were regular on medications as prescribed by the treating physician. Of all the subjects, 81.3% were going for regular consultation. Among them, 86.8 and 89.9% were monitoring their Fasting blood sugar (FBS) and Post prandial blood glucose (PPBS) regularly while only 6.1% monitored HbA1c. One third of the subjects got their Renal function test (RFT) done regularly and 21% underwent yearly ophthalmic evaluation and very meagre proportion of 0.6% underwent regular comprehensive foot assessment. Among the participants who sought regular consultation 57.2, 33.4 and 9.4% were predominantly approaching primary, secondary and tertiary health care facilities respectively.

Most commonly reported co-morbidity was hypertension (64.5%), followed by hypercholesterolemia (17.4%) and IHD (12.6%).

### Lifestyle factors

The most commonly used substance was smokeless tobacco (21.1%), followed by consumption of alcohol (18.1%) and smoking (6.5%). Half of the study participants (51%) were sedentary, 46 and 2.4% were doing light and moderate physical activity. None were involved in heavy physical activity.

Majority (86.5%) of the study participants consumed mixed diet. Among 98.5% people advised on diabetic diet by the treating physician, only 20.3% were following it always and 59.1% were following it most of the times.

### Prevalence of DFS

The overall prevalence of DFS was 51.8% in our population. According to IGWDF Risk Classification, Out of the study population, 48.2% were normal (category 0) while the remaining 51.8% had foot at risk. (Table 1).

**Table 1:** Prevalence of diabetic foot syndrome according to IWGDF Risk Classification System (*n* = 620)

Risk category	Characteristics	*N*	%
Category 0	No peripheral neuropathy	299	48.2
Category 1	Peripheral neuropathy	194	31.4
Category 2	Peripheral neuropathy with peripheral artery disease and/or a foot deformity	74	11.9
Category 3	Peripheral neuropathy and a history of foot ulcer or lower-extremity amputation	53	8.5

About 51.8% subjects had foot at risk, 31.3% had foot at risk category 1; 11.9% patients had foot at risk category 2, in which 10.8% PAD and 10.4% patients had deformity. Only 8.5% of them belonged to category 3, in which 9 had an amputation.

As per MNSI, most common neuropathic symptom perceived by study population was numbness in the feet (51.5%), followed by burning pain (38.7%) and feet being too sensitive to touch (32.9%) and 26.5% subjects responded that symptoms worse at night. Among the participants, 9.8% had a history of previous foot ulceration with 1.5% of them going in for toe amputation.

On inspection of feet, 74.5% of the study subjects had abnormalities in foot appearance which include dry skin (41.9%), deformities (10.5%) including amputation (1.5%), callus (14.5%), infection (15.8%) and ingrown nail (7.6%). Among the study subjects, 1.5% currently presented with foot ulceration. On examination, 45.5% of the study subjects had reduced/absent vibration perception with 128 Hz tuning fork, 34.7% had loss of protective sensation on 10 g SW monofilament testing and 15% had abnormal ankle reflexes. Prevalence of diabetic peripheral neuropathy in the study population was 51.8% based on MNSI examination score.

### Risk factors for DFS

Table 2 describes the association of socio-demographic and lifestyle factors with DFS. Among subjects with DFS, only 5.6% were < 50 years age and increasing proportions of DFS was observed with advancing age of the participants (> 70 years, OR 11.60; 95% CI: 6.06–22.21). The significant association of DFS was observed with low SES (high SES, OR: 0.65; CI: 0.18–2.35), low literacy (Illiterate OR: 2.09; CI: 1.15–3.78), unemployment (OR: 3.04; CI: 1.42–6.49), smokeless tobacco use (OR: 1.82; CI: 1.22–2.71) and sedentary physical activity (OR: 2.29; CI: 0.77–6.78). However other demographic factors like gender and religion did not show any significant association with DFS.

**Table 2:** Univariate analysis for association of socio-demographic and lifestyle factors with diabetic foot syndrome (*n* = 620)

Variables	Diabetic foot syndrome		Unadjusted OR 95% CI	*P* value
	Absent (*n* = 299) *n* (%)	Present (*n* = 321) *n* (%)		
Gender				
Male	116 (38.8)	148 (46.1)	1.35 (0.98–1.85)	0.066
Female	183 (61.2)	173 (53.9)	1	
Age in years				
< 50 years	64 (21.4)	18 (5.6)	1	*< 0.001*
51–60 years	96 (32.1)	62 (19.3)	2.29 (1.24–4.23)	
61–70 years	105 (35.1)	130 (40.5)	4.40 (2.45–7.88)	
> 70 years	34 (11.4)	111 (34.6)	11.60 (6.06–22.21)	
Socio-economic status				
Low	69 (23.1)	105 (32.7)	1	*0.028*
Middle	225 (75.2)	211 (65.7)	0.61 (0.43–0.88)	
High	5 (1.7)	5 (1.6)	0.65 (0.18–2.35)	
Literacy				
Illiterate	36 (12.0)	52 (16.2)	2.09 (1.15–3.78)	*0.018*
Primary	31 (10.4)	51 (15.9)	2.38 (1.29–4.37)	
High school	177 (59.2)	180 (56.1)	1.47 (0.92–2.38)	
PUC and above	55 (18.4)	38 (11.8)	1	
Occupation				
Professional/White collared	29 (9.7)	29 (8.7)	1	*< 0.001*
Skilled/Semiskilled	55 (18.4)	57 (17.8)	1.07 (0.56–2.03)	
Unskilled	13 (4.3)	21 (6.5)	1.67 (0.70–3.97)	
Housewife	152 (50.9)	139 (43.3)	0.94 (0.53–1.67)	
Unemployed	17 (5.7)	50 (15.6)	3.04 (1.42–6.49)	
Retired	33 (11.0)	26 (8.1)	0.81 (0.39–1.69)	
Habits				
Smoking	16 (5.4)	24 (7.5)	1.42 (0.74–2.74)	0.284
Smokeless tobacco use	48 (16.1)	83 (25.9)	1.82 (1.22–2.71)	*0.003*
Alcohol use	49 (16.4)	63 (19.6)	1.24 (0.82–1.88)	0.295
Physical Activity				
Sedentary	116 (38.8)	200 (62.3)	2.29 (0.77–6.78)	*< 0.001*
Light	175 (58.5)	115 (35.8)	0.87 (0.29–2.59)	
Moderate	8 (2.7)	6 (1.9)	1	

*p* < 0.05 is considered to be statistically significant

As depicted in Tables 3 and 4, a significant increase in proportion of DFS was observed among subjects with an increasing duration of DM. Subjects having DM for > 10 years are 3.7 times more likely to develop DFS compared to subjects with duration < 5 years. (OR: 3.77, CI: 2.53–5.62). Univariate analysis revealed presence of IHD (OR: 1.67; CI: 1.02–2.73), use of insulin (OR: 4.50; CI: 1.50–13.47) and level of care (OR: 1.55, CI: 1.09–2.21) was significantly associated with DFS. Anthropometric measurements like BMI, waist circumference; clinical parameters like blood pressure, glycated hemoglobin; presence of comorbidities like hypertension, hypercholesterolemia and health seeking behaviours like adherence to medications, frequency of consulting physician was not significantly associated with DFS.

**Table 3:** Univariate analysis for association of clinical and biochemical parameters with diabetic foot syndrome (N = 620)

Variables	Diabetic foot syndrome		Unadjusted OR 95% CI	*P* value
	Absent (*n* = 299) *n* (%)	Present (*n* = 321) *n* (%)		
Duration of DM				
0–5 years	168 (56.2)	95 (29.5)	1	*< 0.001*
6–10 years	72 (24.1)	100 (31.2)	2.45 (1.65–3.64)	
> 10 years	59 (19.7)	126 (39.3)	3.77 (2.53–5.62)	
Family h/o DM	169 (56.5)	161 (50.2)	0.77 (0.56–1.06)	0.112
Co-morbidities				
Hypertension	187 (62.5)	213 (66.4)	1.18 (0.85–1.64)	0.321
Ischemic heart disease	29 (9.7)	49 (15.3)	1.67 (1.02–2.73)	*0.037*
Hypercholesterolemia	56 (18.7)	52 (16.2)	0.89 (0.55–1.27)	0.407
BMI (kg/m^2^)				
Underweight (< 18.5)	11 (3.7)	13 (4.1)	1	0.736
Normal (18.5–24.9)	141 (47.1)	157 (49.1)	0.94 (0.41–2.18)	
Overweight (25–29.9)	111 (37.1)	106 (33.1)	0.80 (0.34–1.88)	
Obese (≥ 30)	36 (12.1)	44 (13.8)	1.03 (0.41–2.58)	
Waist circumference (cm)				
Normal	72 (24.1)	96 (29.9)	1	0.103
High (Males > 90; Female > 80)	227 (75.9)	225 (70.1)	0.74 (0.52–1.06)	
Blood pressure (mmHg)				
Normal	136 (45.5)	147 (45.8)	1	0.938
High (BP ≥ 140/90)	163 (54.5)	174 (54.2)	0.98 (0.72–1.35)	
HbA1c (mean + SD)^a^ (*n* = 146)	7.7 ± 1.8	7.8 ± 1.8		0.086

^a^Unpaired t-test

*p* < 0.05 is considered to be statistically significant

**Table 4:** Univariate analysis for association of health seeking behaviour with diabetic foot syndrome (*n* = 620)

Variables	Diabetic foot syndrome		Unadjusted OR 95% CI	*P* value
	Absent (*n* = 299) *n* (%)	Present (*n* = 321) *n* (%)		
System of medicine (*n* = 617)				
Allopathy	286 (96.0)	309 (96.9)	1	0.510
Ayurveda	9 (3.0)	5 (1.6)	0.51 (0.17–1.55)	
Both	3 (1.0)	5 (1.6)	1.54 (0.36–6.51)	
Medications for DM (*n* = 603)				
Oral hypoglycemic agents	270 (93.4)	270 (86.0)	1	*< 0.001*
Insulin	4 (5.2)	26 (8.3)	4.50 (1.50–13.47)	
Combined (OHAs+Insulin)	15 (1.4)	18 (5.7)	1.73 (0.89–3.34)	
Adherence to medications^a^				
Yes	286 (95.7)	310 (96.6)	1	0.917
No	13 (4.3)	11 (3.4)	1.28 (0.56–2.90)	
Frequency of consultation^b^				
Regular	246 (82.3)	258 (80.4)	1	0.607
Irregular	53 (17.7)	63 (19.6)	1.13 (0.75–1.69)	
Level of care (*n* = 605)				
Primary	182 (61.7)	105 (52.9)	1	*0.043*
Secondary	84 (28.5)	68 (38.1)	1.55 (1.09–2.21)	
Tertiary	29 (9.8)	12 (9.0)	1.07 (0.61–1.87)	

^a^Adherence to medication: Subject was considered adherent to medication, if he/she is taking prescribed medicines for 6 days or more in a week

^b^Physician consultation was considered regular if he/she is consulting physician once in 3 months or less

*p* < 0.05 is considered to be statistically significant

Table 5 shows multivariate logistic regression analysis for association of factors of diabetic foot syndrome. Advancing age, low socio-economic status, sedentary physical activity and longer duration of DM were significant independent correlates of DFS.

**Table 5:** Multivariate logistic regression analysis for association of factors of diabetic foot syndrome

Variable	Diabetic foot syndrome		Intercept	SE	Wald X^2^	Adjusted OR 95% CI
	Absent (*n* = 299) *n*(%)	Present (*n* = 321) *n*(%)				
Age in years						
< 50 years	64 (21.4)	18 (5.6)			30.56	1
51–60 years	96 (32.1)	62 (19.3)	0.57	0.34	2.79	1.77 (0.90–3.45)
61–70 years	105 (35.1)	130 (40.5)	1.04	0.32	9.50	2.75 (1.44–5.25)
> 70 years	34 (11.4)	111 (34.6)	1.84	0.37	24.67	6.32 (3.05–13.08)
Socio-economic status						
Low	69(23.1)	105 (32.7)			8.40	1
Middle	225 (75.2)	211 (65.7)	−0.60	0.21	8.11	0.54 (0.36–0.82)
High	5 (1.7)	5 (1.6)	−0.81	0.70	1.32	0.44 (0.11–1.77)
Physical Activity						
Sedentary	116 (38.8)	200 (62.3)			11.46	1
Light	175 (58.5)	115 (35.8)	−0.65	0.19	11.37	0.51 (0.35–0.75)
Moderate	8 (2.7)	6 (1.9)	−0.14	0.68	0.47	0.86 (0.22–3.32)
Duration of DM						
0–5 years	168 (56.2)	95 (29.6)			17.74	1
6–10 years	72 (24.1)	100 (31.2)	0.74	0.22	10.51	2.10 (1.34–3.29)
> 10 years	59 (19.7)	126 (39.3)	0.87	0.23	14.59	2.40 (1.53–3.78)

## Discussion

Diabetic foot syndrome is defined as a group of syndromes in which neuropathy, ischemia and infection lead to tissue breakdown, and possible amputation [4]. It is essential to identify the “foot at risk”, through careful inspection and physical examination of the foot followed by neuropathy and vascular tests.

The overall prevalence of DFS was 51.8%. Similar results were observed in a study carried out by Shyam Kishore et al. [17] at Delhi, about 52% patients had foot at risk (category 1 and 2) in which 33 and 19% were in category 1 and 2 respectively.

A study carried out by Lawrence A. Lavery et al. [18], where they have used International Diabetic Foot Classification System which is similar to IWGDF risk classification system. Among 1666 study subjects, 58.6% were in category 0 and 41.4% had foot at risk. Among them, 5.9% had DPN (category 1), 24.7% patients had foot at risk category 2 and about 10.8% of them belonged to category 3. In a study done by Edgar J.G. Peters et al. [19], subjects were stratified as per IWGDF classification and during 3 years of follow-up, ulceration occurred in 5.1, 14.3, 18.8 and 55.8% of the patients in categories 0, 1, 2, and 3, respectively (linear-by-linear association, *P* < 0.001) and all amputations were found in Groups 2 and 3 (3.1 and 20.9%, *P* < 0.001). Thus, it provides evidence that the foot risk classification of the IWGDF foresees ulceration and amputation and can function as a tool to guide prevention of lower extremity complications of diabetes.

DPN is one of the significant components of DFS. Diagnosis of diabetic neuropathy is done through many methods including neurological examination and electrophysiology to detect at its earliest stage.

The MNSI is a rapid, simple and reliable test, validated for Indian settings for screening diabetic peripheral neuropathy in both diabetes clinics and epidemiological surveys [20]. It investigates aspects of both small (pain and hyperesthesia) and large (numbness and muscular) nerve fibre patency. The sensitivity and specificity of MNSI with a cut-off value of 2.5 were 50 and 91%, respectively [21].

Prevalence of diabetic peripheral neuropathy using MNSI in the study population was 51.8%. Similar results were observed in Indian studies done by George H et al. [9] in Tamil Nadu and Mackson Nongmaithem et al. [22] in Maharashtra, where prevalence was found to be 47%. The present study results were similar to studies done outside India, studies done by Rodica Pop-Busui et al. [23] in USA and Gashaw Jember et al. [24] in Ethiopia showed 51 and 52.2% prevalence respectively.

The prevalence of DPN varied from 12 to 60% in different studies done at various parts of India [9, 25–30]. This could be attributed to genetic predisposition, duration of diabetes, existing healthcare facilities, study settings and different diagnostic criteria used.

Advancing age was found to be significantly associated with DFS in many studies in various parts India, Dipika Bansal et al. [25] at Chandigarh, Sailesh K Shahi et al. [31] in Varanasi, Monisha D’Souza et al. [27] in Mangalore, Padmaja Kumari Rani et al. [30], Vijay Viswanathan et al. [32] in Chennai.

A study done by Dipika Bansal et al. [25] showed significant association of low SES with foot complications which are similar to current study. This may be due to lack of awareness, low health seeking behaviour and non-affordability for treatment which makes them prone to develop diabetic complications.

Among subjects with DFS, higher proportions (15.6%) were unemployed compared to subjects without DFS (5.6%). The odds of developing DFS among unskilled workers was 1.67 compared to professionals/white collared job holders and could be ascribed to more exposure to occupational trauma among the former group.

A higher proportion of subjects with DFS were smokers compared to subjects without DFS. Similar observations were made with respect to use of smokeless forms of tobacco and it was significantly associated with DFS. Smoking is an established risk factor for PAD and was identified as a risk factor for DFS in the present study too, in accordance with the research done by Shailesh K Shahi [31] in Varanasi, Mohammad Zubir et al. [33] at Aligarh, Mackson Nongmaithem et al. [22] at Pune, Mamta Jaiswal et al. [34] in USA and Juma M Al-Kaabi et al. [35] in UAE.

Longer duration of diabetes was identified as a risk factor in the study which is in accordance with many neuropathy prevalence studies carried out across the world [25, 26, 31, 35]. BMI and DFS had no significant association. However, obese subjects are 1.03 times more likely to develop DFS compared to underweight subjects (OR 1.03, CI: 0.41–2.58). Similar results were found in studies carried out by Mackson Nongmaithem et al. [22] in Pune and Mamta Jaiswal et al. [34] in USA. Present study did not show any association with HbA1c, this observation is supported in other studies done by Dipika Bansal et al. [25] in north India, RP Agrawal et al. [36] in west India.

Medications taken for the treatment of DM had a significant association with DFS. The subjects with DFS were 4.5 times more likely to be using insulin compared to OHAs (OR 4.50 CI: 1.50–13.47). This could be attributed to the fact that initiation of insulin therapy implies later stages in the natural history of DM. This also correlates with association of DFS with longer duration of DM. Insulin use was associated with severity of DFS in studies conducted by Shilesh K Shahi et al. [31] in north India, Reginald Alex et al. [37], Padmaja Kumari Rani et al. [30] in south India.

The healthcare level the subjects were approaching for treatment showed a significant association with DFS in the present study, similar results are observed in a study carried out by Shyam Kishore et al. [17].

Multivariate logistic regression revealed that advancing age, low socio-economic status, sedentary physical activity and longer duration of DM were significant correlates of DFS.

The strengths of study are; it is a community based study to report the prevalence of DFS while many studies reported prevalence based on hospital patients and we have ensured an adequate sample size and use of validated questionnaire coupled with thorough clinical examination for comprehensive foot evaluation. Limitations of the study include HbA1c not being done for the entire study population due to financial constraints, though we used subset drawn from all DFS risk categories which gives ample evidence about attributes of DFS. Besides DM the study could not assess other co-existing factors which might have led to peripheral neuropathy like autoimmune diseases and nutritional deficiencies as study is done among diabetic patients only. However other causes of neuropathy are considerably less.

## Conclusion

The overall prevalence of diabetic foot syndrome was high among the study population and significantly associated with advancing age, low socio-economic status, sedentary physical activity and longer duration of DM. It can therefore be concluded that the screening for foot complications should start at the time of diagnosis of diabetes and integrated with sustainable patient education at primary care level by training of health care providers at primary care level.

## Abbreviations

ABI: Ankle brachial index
DFS: Diabetic foot syndrome
DM: Diabetes mellitus
DPN: Diabetic peripheral neuropathy
IWGDF: The international working group on the diabetic foot
PAD: Peripheral arterial disease
SES: Socio-economic status
